# Acetosyringone, pH and temperature effects on transient genetic transformation of immature embryos of Brazilian wheat genotypes by *Agrobacterium tumefaciens*

**DOI:** 10.1590/S1415-475738420150026

**Published:** 2015

**Authors:** Ernandes Manfroi, Elene Yamazaki-Lau, Magali F. Grando, Eduardo A. Roesler

**Affiliations:** 1 Departamento de Plantas de Lavoura, Universidade Federal do Rio Grande do Sul, Porto Alegre, RS, Brazil; 2 Centro Nacional de Pesquisa em Trigo, Empresa Brasileira de Pesquisa Agropecuária, Passo Fundo, RS, Brazil; 3 Faculdade de Agronomia e Medicina Veterinária, Universidade de Passo Fundo, Passo Fundo, RS, Brazil

**Keywords:** co-cultivation conditions, protocol establishment, GUS, T-DNA delivery

## Abstract

Low transformation efficiency is one of the main limiting factors in the establishment of genetic transformation of wheat via *Agrobacterium tumefaciens*. To determine more favorable conditions for T-DNA delivery and explant regeneration after infection, this study investigated combinations of acetosyringone concentration and pH variation in the inoculation and co-cultivation media and co-culture temperatures using immature embryos from two Brazilian genotypes (BR 18 Terena and PF 020037). Based on transient expression of *uidA*, the most favorable conditions for T-DNA delivery were culture media with pH 5.0 and 5.4 combined with co-culture temperatures of 22 °C and 25 °C, and a 400 μM acetosyringone supplement. These conditions resulted in blue foci in 81% of the embryos. Media with more acidic pH also presented reduced *A. tumefaciens* overgrowth during co-culture, and improved regeneration frequency of the inoculated explants. BR 18 Terena was more susceptible to infection by *A. tumefaciens* than PF 020037. We found that it is possible to improve T-DNA delivery and explant regeneration by adjusting factors involved in the early stages of *A. tumefaciens* infection. This can contribute to establishing a stable transformation procedure in the future.

Wheat (*Triticum aestivum*) is one of the most cultivated cereal crops worldwide and is a major source of protein and carbohydrates to humans. Nevertheless, like all the crops, it is faced with various biotic and abiotic diseases and other important challenges such as special quality characteristics and improved productivity. These traits can be obtained with the aid of transgenesis (James, 2013).

Several improvements have been made in the wheat transformation protocol since transgenic wheat was first created using *Agrobacterium tumefaciens* in 1997 (Cheng *et al.*, 1997). However, despite a range of protocols that have been developed since then (Cheng *et al.*, 2003,^2004^; Hu *et al.*, 2003; Khanna and Daggard, 2003; Wu *et al.*, 2003; Jones *et al.*, 2005; Patnaik *et al.*, 2006; Agarwal *et al.*, 2009; Ding *et al.*, 2009; Risacher *et al.*, 2009), progress in hexaploid *T. aestivum* has been slower than in tetraploid species (Wu *et al.*, 2008; He *et al.*, 2010) and other cereals such as maize (Vega *et al.*, 2008) and rice (Karthikeyan *et al.*, 2011). Hiei *et al.* (2014) stated that the optimal ranges of many of the factors are narrow in wheat, and that this can be a reason for the slow progress made. The only known protocol giving high stable transformation efficiency is from Japan Tobacco (Ishida *et al.*, 2015), for which use is permitted under license payment.

This difficulty encountered for wheat is due to its huge genome that contains large amounts of repetitive DNA, as well as its low regeneration capacity and resistance to *A. tumefaciens* infection (Bhalla, 2006). Only few studies have analyzed concomitantly the factors related to increased infectivity, such as pH, temperature and acetosyringone (AS) concentration (Gelvin, 2003). It is known that an acid environment and AS act directly on the expression of the *A. tumefaciens vir* region (McCullen and Binns, 2006). Furthermore, temperature is associated with T-pilus formation (Lai *et al.*, 2000). It has been suggested that these factors act together in the beginning of the transformation processes inducing the expression of *vir* genes (He *et al.*, 2010). So far, nothing is known about the liability of Brazilian genotypes for genetic transformation mediated by *A. tumefaciens*, but there are great interests in using genetic material presenting good agronomic traits.

For the establishment of a genetic transformation protocol two main things are: improval of the conditions for T-DNA delivery and the regeneration response of the plant cells. Therefore, we attempted to adjust the factors responsible for the induction of *A. tumefaciens* virulence by determining the best combinations of pH, temperature and phenolic compound (AS) for T-DNA delivery into wheat genomes, as well as its effect on the regeneration of immature embryos subjected to the transformation processes. Two Brazilian wheat genotypes having good agronomic traits were compared with respect to above-mentioned variables. To our knowledge, this work is the first to evaluate concomitantly these main factors related to the early *A. tumefaciens* infection process in wheat.

Donor plants were the Brazilian wheat genotypes PF 020037 and BR 18 Terena. They were grown in 8 L pots in growth chambers with a photoperiod of 16/8 h, a temperature cycle of 21 °C/14 °C (day/night) and light intensity of approximately 150 μmol.m^-2^.s^-1^. Ears were marked at anthesis and harvested after 11 to 14 days, when immature embryos were 0.8 to 1.5 mm long, cream-colored and translucent in appearance. Seeds were disinfested with 70% ethanol (v/v) for 1 min followed by a solution of commercial bleach (2.0 to 2.5% active chlorine) diluted to 1:1 (v/v) in water containing 0.1% (v/v) Tween 20 for 10 min, followed by five rinses with sterile distilled water. Immature embryos were collected aseptically, embryo axes were removed and then placed scutellum-side up in a Petri dish containing co-cultivation medium (Table S1).

*A. tumefaciens* strain AGL1 (Lazo *et al.*, 1991) containing the plasmids pAL154 and pAL156, which is based on pSoup/pGreen vectors (Hellens *et al.*, 2000), was provided by Dr. Huw Jones, Rothamsted Research, UK. The helper plasmid pAL154 contained the 15 kb Komari fragment (Komari, 1990) that provides replication functions for pAL156 in *trans*. The Komari fragment possesses additional *vir* genes (*virG* ^542^, *virB* e *virC*). pAL156 T-DNA contains the *bar* gene (conferring resistance to glufosinate ammonium) and a modified reporter gene, *uidA* gene + intron. Both genes are driven by the Ubi+Intron (maize ubiquitin plus an intron) promoter and the nopaline synthase terminator (*nos*) (Figure S1).

Genetic transformation was performed according to Wu *et al.* (2009), with modifications for the treatments analyzed. Bacteria from glycerol stocks were grown on solid LB medium (10 g L^-1^ Bacto tryptone, 5 g L^-1^ Bacto yeast extract, 5 g L^-1^ NaCl and 15 g L^-1^ agar) supplemented with 100 mg L^-1^ carbenicillin, 50 mg L^-1^ rifampicin and 50 mg L^-1^ kanamycin. One or two streaks of bacterial cells harvested with an inoculation loop were grown in 10 mL of liquid LB medium containing the same antibiotics for 17 to 20 h at 28 °C and 250 rpm. Bacterial cell suspensions were centrifuged at 4,500 x *g* for 10 min. The supernatant was discarded, the pellet resuspended in inoculation medium (Table S1), and the cell concentration was adjusted to an OD_600_ of 1.0 to 2.0. This suspension was supplemented with AS and incubated at 200 rpm and 22 °C for one to three hours. Plates containing approximately 50 embryos received 3 mL of the bacterial suspension supplemented with 0.015% Silwet L77 and were maintained in the dark for 15 min. After removing the bacterial suspension the embryos were transferred to fresh co-cultivation medium and maintained in the dark for three days. Subsequently, the embryos were transferred to induction medium (Table S1). After 3-4 weeks in the dark, the calli were transferred to regeneration medium (Table S1) and grown for an additional three weeks under 30 μmol.m^-2^.s^-1^ light intensity and 16 hours photoperiod. Calli with plantlets were subjected to two rounds of incubation for 3-4 weeks in selective medium (Selection 1 and 2, Table S1).

Expression of the *uidA* gene in transgenic tissues was detected by histochemical test for β-glucuronidase (GUS) (Jefferson *et al.*, 1987). Explants were sampled after 2-3 days on induction medium and incubated at 37 °C for 16 h in X-Gluc buffer [1 mM X-Gluc, 100 mM sodium phosphate buffer pH 7.0, 0.5 mM potassium ferricyanide, 0.5 mM potassium ferrocyanide, and 0.1% (v/v) Triton X-100 with 20% methanol (v/v)]. After this step, the embryos were rinsed with 70% ethanol and stored in the same solution at 4 °C until blue foci were counted. As the counting of GUS foci is very subjective, the same person assessed all experiments, in an attempt to avoid misinterpretations.

Initially, we evaluated the effects of pH, temperature and AS concentration on T-DNA delivery and regeneration of plantlets after infection of immature embryos of the wheat line PF 020037. The factorial experiment (3×3×2) had three pH (5.0, 5.4 and 5.8), three co-cultivation temperature (19, 22 and 25 °C) and two AS concentration levels (200 and 400 μM), and were evaluated using a completely randomized design. Each treatment consisted of approximately 140 immature embryos, with 35 being control embryos (not inoculated) and the remainder inoculated with *A. tumefaciens*. Fifty inoculated embryos were used for histochemical tests, and the remainder proceeded to the subsequent stages of the evaluation protocol. The percentage of embryos with at least one blue focus (% of GUS+) and the number of blue foci per embryo were recorded, and the regeneration frequency (percentage of immature embryos that produced shoots) was determined. This experiment was repeated once. The three best combinations obtained for the wheat line PF 020037 were then used to assess the response of the wheat cultivar BR 18 Terena to T-DNA delivery. The percentage data were transformed by, $c\text{sin}\sqrt{\frac{x}{100}}$ and counting data were transformed by $(\sqrt{x)}+(\sqrt{x+1)}$. Data were subjected to ANOVA for F tests, and means were compared by the Tukey post-hoc test. At p < 0.05 differences were considered as statistically significant.

The three factors studied showed significant interaction (p < 0.05) for both the percentage of GUS+ embryos and the number of blue foci per embryo. Combinations more favorable to infection were seen for media with pH values of 5.0 and 5.4, co-cultivation temperatures of 22 and 25 °C, and, preferably, with the addition of 400 μM AS (Figure 1A-C). Among the media adjusted to a pH of 5.0, the most favorable combinations occurred at 25 °C with 400 μM AS (81.4% GUS+ embryos and 5.7 blue foci per embryo) and at 22 °C with 200 μM AS (71.6% GUS+ embryos and 4.0 blue foci per embryo) (Table 1).

**Figure 1:**
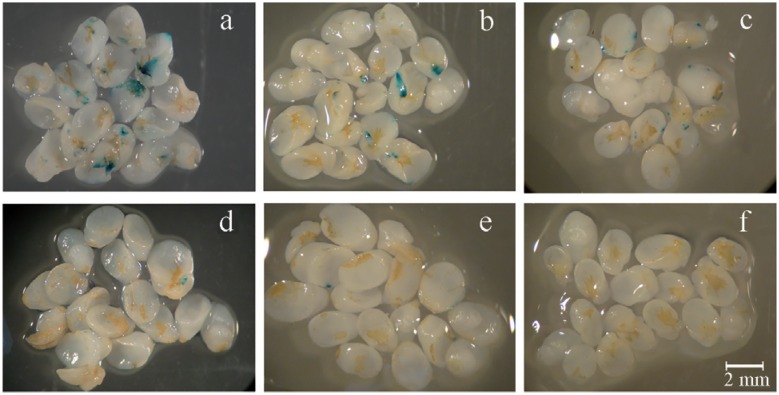
Transient *uidA* (GUS) gene expression in immature embryos of wheat genotype PF 020037 inoculated with *Agrobacterium tumefaciens*. Combinations more favorable for T-DNA expression were: A) pH 5.0 – 22 °C – 200 μM AS; B) pH 5.0 – 25 °C – 400 μM AS;. C) pH 5.4 – 22 °C −400 μM AS. Combinations less favorable for T-DNA expression were: D) pH 5.8 – 19 °C −200 μM AS; E) pH 5.8 – 22 °C −200 μM AS.; F) pH 5.8 – 25 °C – 400 μM AS.

**Table 1:** Effect of the interaction between pH, temperature and acetosyringone (AS) concentration on T-DNA delivery.

Embryos GUS+ (%)						
pH	200 μM AS			400 μM AS		
	19 °C	22 °C	25 °C	19 °C	22 °C	25 °C
5.0	B 44.1^ns^ a	A 71.6* a	AB 50.9* a	B 50.9 a	B 47.1 b	A 81.4 a
5.4	A 43.1^ns^ a	A 39.2* b	A 35.3* a	B 54.9 a	A 77.5 a	AB 55.9 b
5.8	B 8.8^ns^ b	AB 13.7^ns^ c	A 32.4^ns^ a	A 13.7 b	A 13.7 c	A 33.3 c
Number of blue foci per embryo						
pH	200 μM AS			400 μM AS		
	19 °C	22 °C	25 °C	19 °C	22 °C	25 °C
5.0	B 0.9^ns^ a	A 4.0* a	AB 2.1* a	B 1.2 a	B 1.4 b	A 5.7 a
5.4	A 1.1^ns^ a	A 1.2* b	A 1.3* ab	B 1.7 a	A 4.6 a	AB 2.8 b
5.8	A 0.1^ns^ b	A 0.3^ns^ c	A 0.7^ns^ b	A 0.6 b	A 0.3 c	A 0.8 c

Means followed by the same uppercase letter in the row and lowercase letter in the column do not differ, as determined by a Tukey test (p < 0.05). ^ns^ and

* respectively, represent the statistical test results comparing the means for 200 μM and 400 μM AS.

When the inoculation and co-cultivation media were adjusted to a pH of 5.4, the highest rate of T-DNA delivery was obtained at 22 °C with 400 μM AS (77.5% GUS+ embryos and 4.6 blue foci per embryo). Nonetheless, these means were not significantly different from the ones obtained at 25 °C with 400 μM AS (55.9% GUS+ embryos and 2.8 blue foci per embryo). Culture media adjusted to pH 5.8 did not provide favorable combinations for infection (Figure 1D-F) and yielded lower infection frequencies than pH values of 5.0 and 5.4 for all combinations of temperature and AS concentration, with the exception of 25 °C and 200 μM AS.

Considering the four treatments that showed significant differences between the two AS concentrations investigated, culture media containing 400 μM resulted in higher T-DNA delivery at three different pH and temperature combinations (pH 5.0 −25 °C; pH 5.4 −22 °C and pH 5.4 −25 °C). The best treatment when using 200 μM AS was in a combination of pH of 5.0 with 22 °C. Co-cultivation temperatures of 22 and 25 °C were more favorable for infection, except for combinations of pH 5.4 and 200 μM AS, and for pH 5.8 and 400 μM AS. These two combinations showed equal means at the three temperatures investigated (Table 1).

The two most recent protocols relevant for wheat transformation used 400 μM (Sparks *et al.*, 2014) and 100 μM AS (Ishida *et al.*, 2015), respectively, both using Fielder. Sparks *et al.* (2014) suggest the use of 200 μM to 800 μM AS. Thus, even though the AS concentration is important, it does not seem to be the main factor that needs to be optimized, as the most efficient protocol published by Ishida *et al.* (2015) uses only 100 μM.

The overgrowth of *A. tumefaciens* was more pronounced when pH and temperature were increased (Table 2). The regeneration frequency of control explants was greater than for the inoculated ones for all combinations, and it remained stable regardless of pH. Therefore, the immature embryos were probably damaged by *A. tumefaciens* overgrowth during co-cultivation and not by the culture media condition itself. Among all combinations analyzed, regeneration frequencies ranged from 23.1 to 58.1% for the control and from 0 to 31% for the inoculated explants. *A. tumefaciens* overgrowth in media with a pH 5.8 correlated with reduced explant regeneration capacity. The average loss of capacity to regenerate in these media was of 89.1%, which is far higher than the 24.9% average loss in media with a pH of 5.0 (data not shown). No relevant losses were observed when comparing the two AS concentrations. The transient transformation increased when using a 400 μM AS concentration at pH 5.4, but explant regeneration capacity decreased. Similar behavior was, however, not observed in media with a pH of 5.0. Although some plants survived the selection step, no transgenic plants were confirmed by PCR targeting the *bar* and *uidA* genes. We suspect that the phosphinothricin (PPT) concentration used in the selection medium was not sufficient to prevent the survival of non-transgenic plants. Increasing this concentration to 10 mg L^-1^, as used by Ishida *et al.* (2015), may reduce this problem. Also, it was observed that *A. tumefaciens* prefers to infect scutellar borders, which are considered less likely for callus initiation (Ke *et al.*, 2002; McCormac *et al.*, 1998) than wound regions, which could be another reason for not obtaining transgenic plants.

**Table 2:** Regeneration frequency of wheat immature embryos (PF 020037 genotype) not inoculated (control) and inoculated with *A. tumefaciens,* as well as bacteria overgrowth levels during co-cultivation.

Treatments			Regeneration frequency (%)		*A. tumefaciens* overgrowth during co-cultivation
pH	Temperature (°C)	AS (μM)	Control	Inoculated	
5.0	19	200	32.6	24.8	–
		400	37.8	30.3	–
	22	200	35.4	22.3	–
		400	29.7	24.5	–
	25	200	44.7	28.1	–
		400	30.5	28.0	–
5.4	19	200	42.1	23.8	–
		400	35.3	14.4	–
	22	200	32.2	12.2	+
		400	37.1	7.5	+
	25	200	37.0	31.3	+
		400	58.1	17.6	+
5.8	19	200	52.5	5.0	+
		400	37.5	0	++
	22	200	23.1	10.5	++
		400	33.3	0	++
	25	200	55.8	0	+++
		400	32.0	10	+++

The three best combinations found in the first experiment [(I) pH 5.0 – 25 °C – 400 μM AS, (II) pH 5.4 −22 °C – 400 μM AS e (III) pH 5.0 −22 °C – 200 μM AS] were the treatments used to compare the response to GUS expression in BR 18 Terena and PF 020037. There was no interaction between combinations (I, II, and III) regarding genotypes (p > 0.05) and there was no significant difference between them for the percentage of GUS+ embryos and the number of blue foci per embryo (Table 3). However, BR 18 Terena was superior, as the percentage of GUS+ embryos of 71.8% as 13% higher than the one observed in PF 020037 (58.8%). For the number of blue foci per embryo the two wheat genotypes were similar to GUS+ response (5.2 in BR 18 Terena and 2.8 in PF 020037).

**Table 3:** Effect of wheat genotype on T-DNA delivery.

Treatments^1^	Genotype		Mean
	PF 020037	BR 18 Terena	
Embryos GUS+ (%)			
I	52.9 ± 6.8^2^	75.3 ± 7.4	64.1 a
II	60.8 ± 3.9	74.0 ± 8.1	67.4 a
III	62.7 ± 5.2	66.1 ± 3.6	64.4 a
Mean	58.8 B	71.8 A	
Number of blue foci per embryo			
I	3.7 ± 0.7	6.2 ± 0.5	4.9 a
II	2.5 ± 0.3	4.7 ± 0.9	3.6 a
III	2.3 ± 0.2	4.7 ± 1.0	3.5 a
Mean	2.8 B	5.2 A	

Means followed by same uppercase letter in the row and lowercase letter in the column do not differ, as determined by a Tukey test (p < 0.05).

1 I (medium with pH 5.0 – 25 °C – 400 μM AS); II (medium with pH 5.4 −22 °C – 400 μM AS); III (medium with pH 5.0 −22 °C – 200 μM AS).

2 mean ± standard error of the mean.

The basic requirement for a successful gene transfer system is the ability to transfer an intact DNA segment into the nuclear genome of regenerable cells and the ability to recover adult plants from transformed cells via tissue culture (Jones, 2005). Our work shows that it is possible to improve T-DNA delivery into plant cell genomes by adjusting the factors responsible for inducing the *vir* region, such as pH, temperature and AS concentration (McCullen and Binns, 2006).

Our results (Table 1, Figure 1) support the hypothesis that AS interacts with specific temperatures and acidic environments to promote the expression of *vir* genes in *A. tumefaciens* during the inoculation and co-cultivation period (He *et al.*, 2010). The majority of published wheat genetic transformation protocols report pH values of 5.7 – 5.8 for the culture medium (Cheng *et al.*, 2004; Jones *et al.*, 2005; Wu *et al.*, 2009). However, more acidic pHs (5.2-5.4) are described in the transformation protocols of other monocots such as maize (Frame *et al.*, 2006; Vega *et al.*, 2008) and rice (Hiei and Komari, 2006; Patel *et al.*, 2013). At pH 5.0, a strong trend towards increased T-DNA delivery with increasing AS concentration was observed without any negative effects on the regeneration potential of immature embryos. However, at pH 5.4, regeneration capacity was affected when T-DNA delivery was increased by increasing the AS concentration. He *et al.* (2010) reported that increasing transient transformation by increasing AS concentration from 200 to 400 μM did not decrease the regeneration frequency of somatic embryos; however, this work was carried out using *T. durum* and at pH of 5.8. As bacterial overgrowth occurs in treatments utilizing culture media with pH 5.4, embryo tissues have probably been sufficiently damaged to compromise the regeneration but not the transformation and expression of the T-DNA. This is an example for the claim by Hiei *et al.* (2014), that for wheat the optimal ranges of many of the factors for transformability are narrow.

In this study, we observed, by visual analysis, that the occurrence of overgrowth by *A. tumefaciens* is less during co-cultivation in culture media with pH 5.0 than with those of pH 5.4 and 5.8. Fewer bacteria likely represent reduced damage to embryo tissues, resulting in better plantlet regeneration. Wu *et al.* (2009) reported that *A. tumefaciens* overgrowth during co-cultivation is a problem for the regeneration of immature embryo calluses when using the same strain and inoculation and similar co-cultivation medium pH (5.7). An alternative to decreasing *A. tumefaciens* overgrowth during co-culture may be to reduce co-cultivation time from three to two days. However, care must be taken, as this reduction may affect T-DNA expression. To overcome the overgrowth problem Khanna and Daggard (2003) decreased the temperature after the first day of co-culture. These authors also included spermidine in the induction medium to facilitate the recuperation of infected tissues. Lowering the bacterial concentration or rinsing the explants with liquid culture medium, has also been recommended (Hensel *et al.*, 2009).

The Brazilian wheat cultivar BR 18 Terena demonstrated good potential for genetic transformation, as it is more susceptible to *A. tumefaciens* than PF 020037. Both genotypes were used because they showed ability to regenerate and capability of transient transformation in previous experiment. In addition, under the same assay conditions, the model genotype Bobwhite had very low levels of transient transformation. This finding probably correlates with the higher adaptability of the Brazilian genotypes to the growth conditions of the donor plants. The fact that BR 18 Terena is an elite wheat cultivar should facilitate the introduction of transgenes into breeding programs. In conclusion, the joint analysis of key factors, such as pH, temperature and AS, involved in the early stages of *A. tumefaciens*-mediated transformation revealed that such factors are not simply additive and can be manipulated to improve the efficiency of T-DNA delivery and to control bacterial overgrowth during co-cultivation.

## Supplementary Material

The following online material is available for this article:

Figure S1 - Schematic map of the T-DNA region of the pAL156 vector

Table S1 - Culture medium composition

This material is available as part of the online article from: http://www.scielo.br/gmb.
