# Supplementary material for: Acetosyringone, pH and temperature effects on transient genetic transformation of immature embryos of Brazilian wheat genotypes by Agrobacterium tumefaciens
Source: Genet Mol Biol. 2015 Oct-Dec;38(4):470–6. doi: 10.1590/S1415-475738420150026 (PMC4763325; doi:10.1590/S1415-475738420150026)
Supplement: Figure S1 - [file 1415-4757-gmb-S1415-475738420150026-s001.pdf]

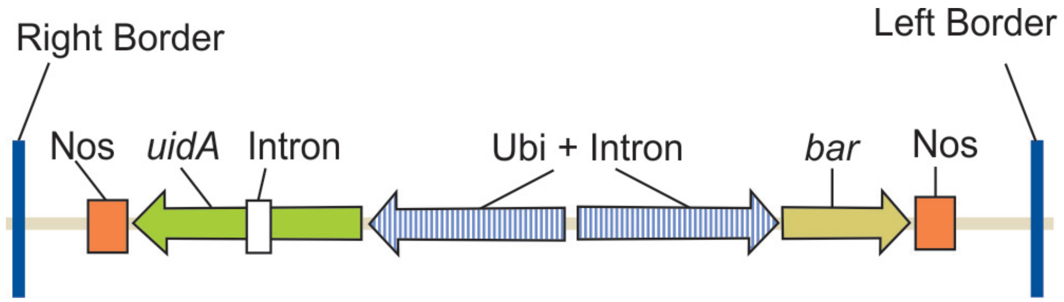

Figure S1 - Schematic map of the T-DNA region of the pAL156 vector showing the construction with the modified *uidA* gene (uidA+Intron) and the *bar* gene. Both are driven by the Ubi+Intron promoter. Nos - nopaline synthase terminator.
