# Supplementary material for: Acetosyringone, pH and temperature effects on transient genetic transformation of immature embryos of Brazilian wheat genotypes by Agrobacterium tumefaciens
Source: Genet Mol Biol. 2015 Oct-Dec;38(4):470–6. doi: 10.1590/S1415-475738420150026 (PMC4763325; doi:10.1590/S1415-475738420150026)
Supplement: Table S1 - [file 1415-4757-gmb-S1415-475738420150026-s002.pdf]

Table S1 - Culture medium composition.

Medium type	Composition
Inoculation	1/10 Macronutrients MS[1], 1/10 Micronutrients L7[1], 10 mL L^{-1} $FeNa_2$ EDTA, 10 mL L^{-1} Vitamins MS[1], 100 mg L^{-1} casein hydrolysate, 1.95 g L^{-1} MES, 100 mg L^{-1} inositol, 10 g L^{-1} glucose, 40 g L^{-1} maltose, 2 mg L^{-1} picloram, 2 mg L^{-1} 2,4-D, 200 or 400 µM acetosyringone, pH (5.0, 5.4 or 5.8)
Co-cultivation	Same composition as inoculation medium, but solidified with 2 g L^{-1} phytagel and full salt (macro and micronutrients) strength
Induction	Same composition as inoculation medium, but without glucose and acetosyringone. Addition of 1.03 g L^{-1} $CaCl_2.2H_2O$, 1.25 mg L^{-1} $CuSo_4.5H_2O$, 500 mg L^{-1} glutamine, 0.5 mg L^{-1} 2,4-D, 160 mg L^{-1} Timentin and 5 g L^{-1} agargel, pH 5.7
Regeneration	Macronutrients L7[1], micronutrients L7, vitamins/inositol[1], 10 mL L^{-1} FeNa2 EDTA, 100 mg L^{-1} inositol, 30 g L^{-1} maltose, 1 mg L^{-1} zeatin, 0,1 mg L^{-1} 2,4-D, 160 mg L^{-1} Timentin, 5 g L^{-1} agargel, pH 5.7
Selection 1	Same composition of regeneration medium, but without zeatin and with 3 mg L^{-1} phosphinothricin (PPT) added
Selection 2	Same as Selection 1 but with 4 mg L^{-1} PPT.

[1] Stock solutions described in Wu *et al.* (2009).
